# Mental Health Support in Assisted Reproductive Technology Centers

**DOI:** 10.1192/j.eurpsy.2025.1568

**Published:** 2025-08-26

**Authors:** N. Bouayed Abdelmoula

**Affiliations:** 1 Genomics of Signalopathies at the service of Precision Medicine, LR23ES07, Medical University of Sfax, Sfax, Tunisia

## Abstract

**Introduction:**

Access to genetic testing and counseling for infertile couples is essential to provide them specialized care at the genetic level. Having access to psychological support during genetic fertility assessment can be very helpful for many patient and genetic counseling offers invaluable emotional support throughout the process.

**Objectives:**

Based on our twenty-four years’ experience in genetic counseling, we present here the main psychological challenges facing couples struggling with fertility problems.

**Methods:**

We selected all infertile couples evaluated between 2000 and 2024, who were treating their infertility with assisted reproductive technologies. Patients’ psychological problems and emotional suffering, as well as the causes of these conditions, were evaluated retrospectively through our genetic counseling reports.

**Results:**

All couples suffering from primary infertility show a more obvious decline in general well-being and satisfaction as a couple, when they are compared to couples seeking ART for secondary infertility, but having already other children. Anxiety, depression and feelings of culpability in infertile partners are the most common psychological concerns. Besides the psychological distress related to infertility, ART itself is, despite the hope it offers in terms of parenthood prognosis, a stress factor and a cause of numerous emotional and psychological problems. This distress is related to the invasive features of the ART, such as intra-testicular sperm extraction in azoospermic men and oocyte puncture, and the hormonal use in the female partners, which disturb their behaviors and moods. Other problems are associated with the lengthy, arduous and economically burdensome procedures involved in the entire treatment process. In addition, the worries of failure and the distress of failure when it becomes a reality are a crucial factor in psychological distress for the couple. Furthermore, dissatisfaction and frustration when ART is undergone for infertility affecting only one partner were noted. Many religious issues and worries about fear or phobia concerning germ cell manipulation also contribute to psychological problems. Genetic testing is another burden for the couples in our study, given the widespread ignorance of genetics and the consequent phobia of the unknown, as well as the fear of results that, once revealed as abnormal, will affect self-esteem for life.

**Conclusions:**

This study describe the psychological concers of infertile couples undergoing ART treatment in the Tunisian Context. The improvement of the understanding of psychological needs, which are certainly provided during genetic counselling, requires a multidisciplinary consultation, involving gynecologist, reproductive biologists, geneticists, psychiatrists and psychologists. This would enable holistic and personalized psychological intervention and support.

**Disclosure of Interest:**

None Declared

